# Screen-Printable Silver Paste Material for Semitransparent and Flexible Metal–Semiconductor–Metal Photodetectors with Liquid-Phase Procedure

**DOI:** 10.3390/nano12142428

**Published:** 2022-07-15

**Authors:** Shang Yu Tsai, Ching-Chang Lin, Cheng-Tang Yu, Yen-Shuo Chen, Wei-Lin Wu, Yu-Cheng Chang, Chun Chi Chen, Fu-Hsiang Ko

**Affiliations:** 1 Department of Materials Science and Engineering, National Yang Ming Chiao Tung University, 1001 University Road, Hsinchu 30010, Taiwan; ntesst6105@gmail.com (S.Y.T.); tom568899@gmail.com (C.-T.Y.); rubioibur00@gmail.com (Y.-S.C.); lin110489@gmail.com (W.-L.W.); 2 Research Center for Advanced Science and Technology (RCAST), The University of Tokyo, 4-6-1 Komaba, Meguro-ku, Tokyo 153-8904, Japan; lin@dsc.rcast.u-tokyo.ac.jp; 3 Department of Materials Science and Engineering, Feng Chia University, Taichung 407, Taiwan; yuchchang@fcu.edu.tw; 4 Taiwan Semiconductor Research Institute, 26, Prosperity Road I, Hsinchu Science Park, Hsinchu 300091, Taiwan; chunchi.chen@narlabs.org.tw

**Keywords:** metal–semiconductor–metal, visible light photodetector, flexible, semitransparent silver paste electrodes, screen printing

## Abstract

Photodetectors are widely applied in modern industrial fields because they convert light energy into electrical signals. We propose a printable silver (Ag) paste electrode for a highly flexible metal–semiconductor–metal (MSM) broadband visible light photodetector as a wearable and portable device. Single-crystal and surface-textured silicon substrates with thicknesses of 37.21 μm were fabricated using a wet etching process. Surface texturization on flexible Si substrates enhances the light-trapping effect and minimizes reflectance from the incident light, and the average reflectance is reduced by 16.3% with pyramid-like structures. In this study, semitransparent, conductive Ag paste electrodes were manufactured using a screen-printing with liquid-phase process to form a flexible MSM broadband visible light photodetector. The transmittance of the homemade Ag paste solution fell between 34.83% and 36.98% in the wavelength range of visible light, from 400 nm to 800 nm. The highest visible light photosensitivity was 1.75 × 10^4^ at 19.5 W/m^2^. The photocurrents of the flexible MSM broadband visible light photodetector were slightly changed under concave and convex conditions, displaying stable and durable bending properties.

## 1. Introduction

Currently, detectors are indispensable in modern life. The principle of detectors is to convert specific energies into other types of signals that can be read by an observer. An interesting category of detectors is photodetectors, which convert light energy into electrical signals [1,2,3,4,5,6,7,8]. According to the wavelength of the detection range, photodetectors are applied to detect ultraviolet (UV), infrared (IR), and visible regions [9,10,11,12,13,14]. In particular, the wavelength of visible light is approximately located in the range of 380 nm to 780 nm. Visible light is important in our living environments because of the sensitivity of the human eye to visible light; thus, visible light photodetectors are one of the most popular applications. To detect light effectively, the photodetectors must have a fast response and higher sensitivity. Photodiodes such as P-N junctions and P-I-N structures are popular types of photodetectors. However, such structures display low photoresponsivity and need to boost the electrical signals via amplifiers [15,16]. The P-N junction design exhibits the low leakage current property. In contrast, the structure of the metal–semiconductor Schottky contact demonstrates various advantages such as high switching frequency, low forward bias voltage, and narrow depletion region. In comparison, metal–semiconductor–metal (MSM) photodetectors with two metal–semiconductor Schottky contacts have faster light detection capabilities and higher bandwidths [17,18].

Flexible photodetectors may become the next generation of optoelectronic devices owing to their portable and wearable properties, such as bionic imaging, mobile devices, image sensors, and eye cameras [19,20,21,22,23,24]. Silicon (Si) is a promising material in modern industrial optoelectronics for fabricating flexible photodetectors. Researchers have been devoted to studying Si-based flexible photodetectors, such as nanostructures and heterojunctions with Si [25,26,27,28,29]. To fabricate the flexible, conductive electrodes, the printing processes made it possible to deposit conductive thin films on flexible substrates [30]. Because the screen-printing process does not require an expensive vacuum system, the process is a cost-effective printing technique to fabricate flexible, conductive electrodes [31]. Furthermore, the advantages of the screen-printing process are that it is simple, rapidly fabricated, and produced in large quantities. The process is also applied on any shape surface, such as plastics, glass, or clothes [32,33]. For fabricating electrodes with a screen-printing process, the conductive materials of the printed ink are chosen to be smaller than the mesh. The conductive materials of ink include carbon nanotubes, silver powders, and silver nanowires [34,35,36,37,38,39]. R. Faddoul et al. synthesized silver flake pastes and successfully screen-printed them on low-temperature cold-fired ceramic (LTCC) substrates [30]. The electrical resistivity of the ink varied from 1.6 × 10^−8^ to 3.3 × 10^−8^ Ω-m, which was in the same order of magnitude as bulk silver (1.58 × 10^−8^ Ω-m). H. Lan et al. fabricated nanosilver paste on a glass substrate via electric-field-driven (EFD) micro-scale 3D printing technology. Although the nanosilver paste displayed better sheet resistance of 1.48 Ω/sq, the fabrication process needed a high-voltage power supply [40]. The photosensitivity should remain in the bending condition for a stable flexible photodetector. Hence, flexible, conductive electrodes must maintain electricity and light transmittance at any bending angle.

In this study, two important methods were proposed to fabricate highly sensitive and durable bending flexible MSM broadband visible light photodetectors. The first issue was to lower the reflectance of Si substrates. Flexible Si with a textured substrate was fabricated using wet etching sodium hydroxide (NaOH) solution, and pyramid-like structures were shown to exist on the surface of flexible Si. The textured surface enhanced the light-trapping ability and effectively decreased the reflection rate of the incident light. The ultraviolet/visible/near infrared (UV/VIS/NIR) spectrophotometer results display a lower average reflectance of 20.76% on flexible Si with pyramid-like structures. The second issue was the fabrication of printable, semitransparent, and Schottky contact electrodes on flexible Si with textured substrates to form MSM structures. Conductive Ag paste electrodes were screen-printed on flexible Si with pyramid-like structures. The advantage of semitransparent Ag paste electrodes is that they increase the transparency of the incident light and effectively raise the ability of photo-to-electrical transformation. The highest visible light photosensitivity was 1.75 × 10^4^ at 19.5 W/m^2^. The photocurrents of the flexible MSM broadband visible light photodetector were slightly changed under concave and convex conditions, indicating stable and durable bending properties.

## 2. Materials and Methods

### 2.1. Flexible and Surface Texturization of Si Substrate Fabrication

A 6-inch P-type silicon (100) wafer was cut into 9 cm^2^ square substrates. The square substrates were sequentially cleaned with acetone, ethanol, and deionized (DI) water (18 MΩ·cm^−1^) and then dried with nitrogen gas to remove moisture. To fabricate a flexible Si substrate, square Si substrates were separated into several segments by microslides and immersed in a 40 wt % sodium hydroxide (NaOH) solution with a capped vessel. The temperature of the NaOH solution was maintained at 70 °C during the etching process by a thermostat oil batch (EYELA PS-1000, EYELA Co., Tokyo, Japan) with a magnetic stirrer. The above immersion sequence in NaOH solution is called the wet etching process. The etching times of the wet etching process were 1, 2, 3, 4, 5, and 6 h.

**Figure 1:**
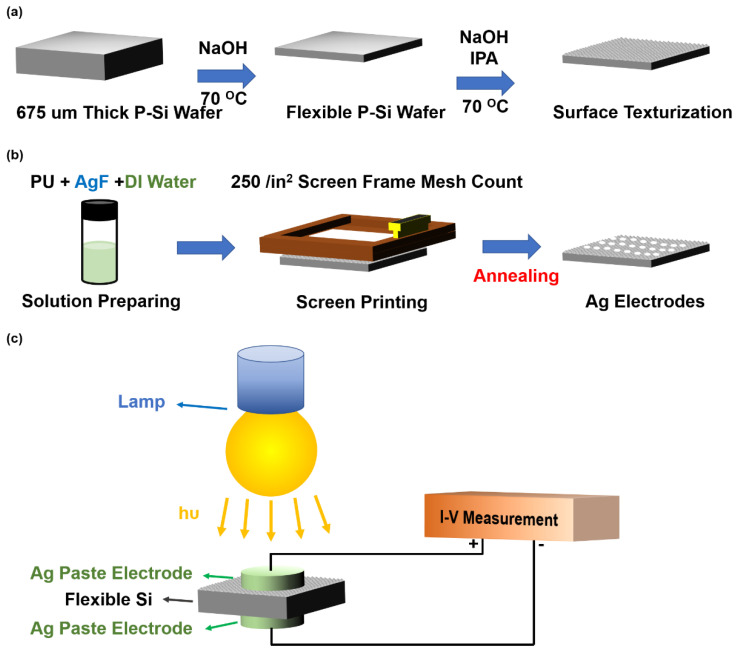
The fabrication process of (**a**) flexible Si with surface texturization and (**b**) Ag electrodes with the screen-printing method. (**c**) Schematic diagram of the photoelectric measurement system with the MSM structure. The electrical properties of the MSM structure were measured using the probe system, and the incident light power was controlled by the lamp.

After the etching process of Si substrates, the flexible Si substrates were also sequentially cleaned with the abovementioned cleaning process. To manufacture the surface texturization on flexible Si substrates, the substrates were immersed in 5 wt % NaOH solution and 5% *v/v* isopropyl alcohol (IPA) at 70 °C for 30 min in a thermostatted oil bath with magnetic stirring. The fabrication process flow of flexible Si with surface texturization is illustrated in Figure 1a.

### 2.2. Silver Paste MSM Broadband Visible Light Photodetector Fabrication

To fabricate the MSM visible light photodetector with Schottky contact, silver (Ag) metal was chosen as the top and back electrodes. Here, we fabricated printable, semitransparent, conductive Ag paste as top electrodes on flexible and surface-texturized P-type Si substrates to enhance visible light absorption and sensitivity. Conductive Ag paste was utilized as the 250/in^2^ meshed electrode in the screen-printing process. To fabricate printable, semitransparent, conductive Ag paste, polyurethane (PU), AgF, and DI water were mixed. The mixed Ag paste solution was ultrasonicated with an ultrasonicator for 10 min and then stirred for 1 h with a magnetic stirrer. The concentrations of PU and the AgF solution ratio were fixed at 1:4, and the amount of DI water solvent was 0.5, 1, 1.5, and 2. The respective DI water solvents of various Ag paste solutions were numbered 8005, 8010, 8015, and 8020, as illustrated in Table 1. Then, various Ag paste solutions were screen-printed on the 250/in^2^ meshed electrodes. After fabricating the top electrodes, the 250/in^2^ meshed Ag paste electrodes were annealed at 160 °C for 30 min. The fabrication process of the MSM broadband visible light photodetector is displayed in Figure 1b.

### 2.3. Characteristics

Morphology measurements were observed using scanning electron microscopy (SEM, JEOL 6700; JEOL, Tokyo, Japan). The crystallinity was found using X-ray diffraction (XRD, D8 DISCOVER, Bruker Co., Billerica, MA, USA) instruments with an incident X-ray wavelength of 1.5418 Å. The optical properties of MSM structures were determined with a variable-angle UV/VIS/NIR spectrophotometer. The electrical properties of MSM structures were measured with a Keysight B1500A (Keysight, Santa Rosa, CA, USA). The photoelectric measurement system of the MSM structures is illustrated in Figure 1c.

## 3. Results and Discussion

### 3.1. Flexibility and Surface Texturization of Si

**Figure 2:**
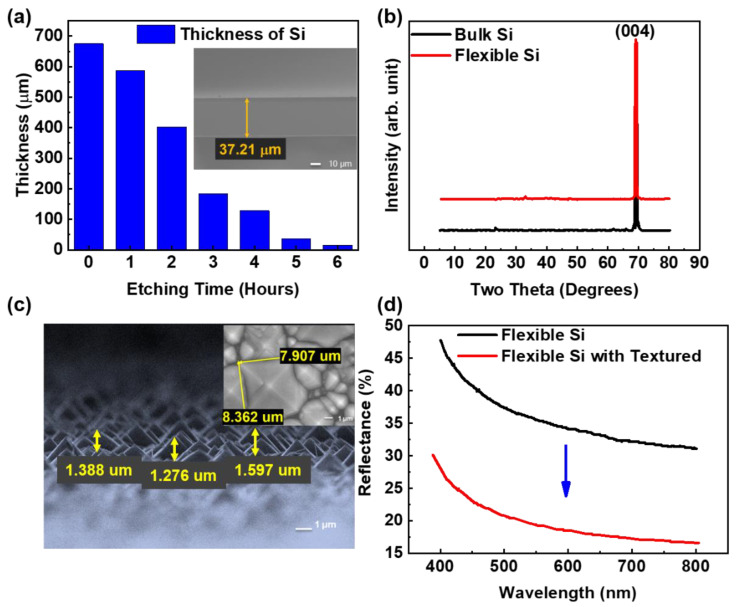
(**a**) The thickness of Si substrates with increasing etching time, (**b**) the XRD results of Si substrates with thicknesses of 675 μm (bulk) and 37.21 μm (flexibility), (**c**) the SEM images of flexible Si substrates with textured structures after NaOH and IPA solution immersion at 70 °C for 30 min, and (**d**) the reflectance of flexible Si with and without surface texturization.

Generally, the thickness of the 6-inch bulk P-type Si substrate was 675 μm, and the substrate was rigid and inflexible. To achieve the flexibility of the Si substrate, the wet etching process was utilized to cut the thickness of the Si substrate. As the thickness of the Si substrate approached 30 μm, the substrate attained flexibility [41]. Traditionally, chemical-mechanical planarization (CMP) process has been utilized to slim the thickness of silicon substrate. The surface of the silicon substrate is flat after the expensive CMP process, and the reflectivity of the incident light is high. Our solution approach could avoid using expensive tools, and it obtained a textured and flexible silicon surface. Figure 2a displays the thickness of Si substrates with increasing etching time from 1 to 6 h. The results indicated that the thicknesses of the Si substrates were 586.9 µm, 403.1 μm, 183.8 μm, 128.0 μm, 37.21 μm, and 15.55 μm, with etching times of 1, 2, 3, 4, 5, and 6 h, respectively. These results showed that the thickness of Si substrates decreased with increasing etching time during the wet etching process, indicating the controllable thickness of Si substrates with the etching time in the wet etching process. The thickness of Si substrates with increased etching time was observed in the cross-section SEM images, as shown in Figure S1. These results displayed the uniform thickness of Si substrates during the wet etching process. Considering the following etching steps for surface texturization, Si substrates with thicknesses of 37.21 μm with 5 h of etching were chosen in our study. To check the crystallinity of Si substrates with the etching process, XRD was utilized. Figure 2b indicates the XRD results of Si substrates with thicknesses of 675 μm and 37.21 μm. The thicknesses of 675 μm and 37.21 μm were not etched and were etched for 5 h on Si substrates, respectively. The results displayed only the (004) peak located at 69.13° in both Si substrates, indicating that the crystal structure of the Si substrates was nondestructive after the wet etching process.

To effectively improve the conversion efficiency from visible light to electricity to enhance the sensitivity of broadband visible light photodetectors, the surface texturization of Si substrates was fabricated. Here, Si substrates with a thickness of 37.21 μm were chosen for immersion in a solution of 5 wt % NaOH and 5% *v/v* IPA at 70 °C for 30 min to fabricate surface-textured Si substrates. Figure 2c displays the SEM images of flexible Si substrates with textured structures after NaOH and IPA solution immersion at 70 °C for 30 min. The results indicated that uniform pyramid-like structures were observed on the surface of flexible Si substrates. The height of the pyramid-like Si structures decreased between 1.2 μm and 1.6 μm. The inset of Figure 2c shows that the length and width of the pyramid-like Si structures were 8.4 μm and 7.9 μm, respectively. To observe the reflectance of flexible Si substrates with pyramid-like structures, a UV/VIS/NIR spectrophotometer was used. Figure 2d indicates the reflectance of flexible Si with and without surface texturization. The results indicated that the average reflectance of flexible Si with and without surface texturization was 20.76% and 37.06%, respectively. The results showed that the reflectance of flexible Si with pyramid-like structures was reduced in the range from 400 nm to 800 nm, indicating the enhanced light-trapping effect of visible light by pyramid-like structures. Therefore, the reflectance of incident visible light was effectively reduced.

### 3.2. Electrical and Optical Properties of Conductive Silver Paste

**Figure 3:**
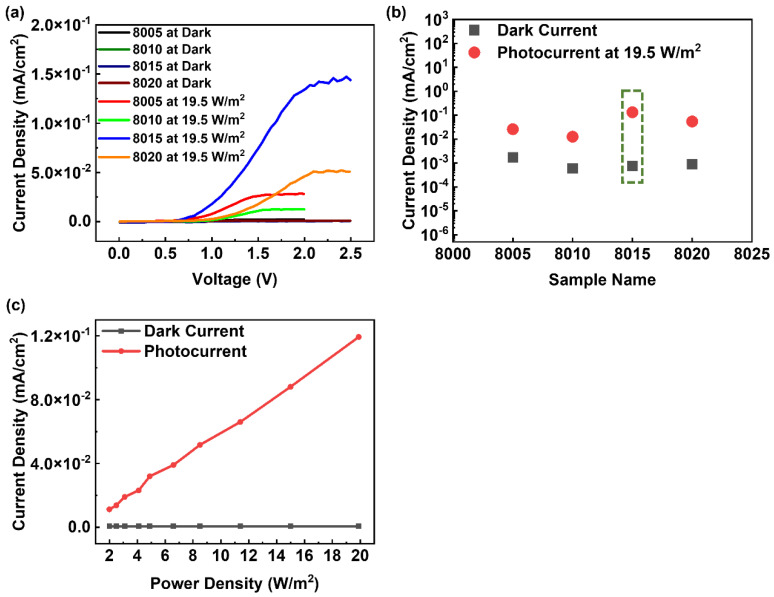
(**a**) The I-V characteristics of MSM broadband visible light photodetectors with different homemade conductive materials. (**b**) The current density of the flexible MSM broadband visible light photodetector with different numbered conductive Ag paste electrodes. (**c**) The current density of the flexible MSM broadband visible light photodetector with homemade numbered 8015 conductive Ag paste electrodes under different light power densities.

**Figure 4:**
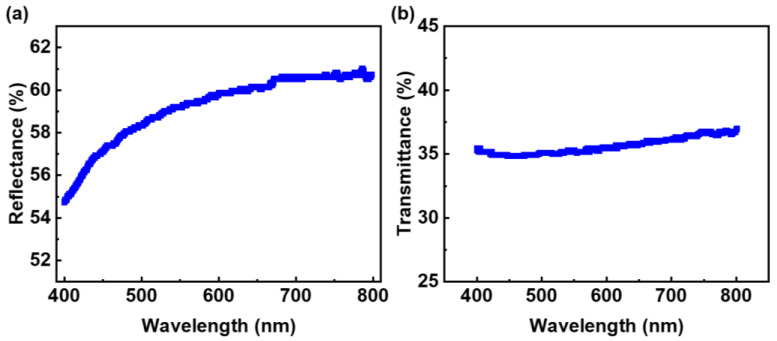
(**a**) The reflectance and (**b**) transmittance of homemade numbered 8015 conductive Ag paste solution.

To fabricate the flexible MSM broadband visible light photodetector, semitransparent, conductive Ag paste electrodes were manufactured on flexible and surface-textured Si substrates using a screen-printing process. The semitransparent, conductive Ag paste was synthesized by mixing PU, AgF, and DI water. The concentrations of PU and AgF solution ratio were fixed at 1:4, and the concentrations of DI water solvents were varied from 0.5 to 2. Here, the different DI water concentrations of 0.5, 1, 1.5, and 2 with PU and AgF homemade paste solutions were numbered 8005, 8010, 8015, and 8020, respectively (see Table 1). To discuss the sensitivity of the flexible MSM broadband visible light photodetector with conductive Ag paste electrodes in different DI water concentrations, the I-V characterization is shown in Figure 3a. Figure 3b indicates the current density of the flexible MSM broadband visible light photodetectors in the dark and 19.5 W/m^2^ visible light with conductive Ag paste electrodes in different DI water concentrations. The photo currents and dark currents of the flexible MSM broadband visible light photodetectors with conductive Ag paste electrodes in different DI water concentrations were derived from Figure 3a. The increased current density under 19.5 W/m^2^ visible light indicated the photoresponse of visible light. The photosensitivity was derived by the formula shown below [42,43,44]:

(1) $$S=\frac{I_{P}-I_{D}}{I_{D}}\times 100\%$$

where S is the sensitivity to visible light, *I_P_* is the photocurrent density, and *I_D_* is the dark current density. The sensitivity of the flexible MSM visible light photodetectors under 19.5 W/m^2^ was 1.38 × 10^3^, 2.03 × 10^3^, 1.75 × 10^4^, and 6.13 × 10^3^, corresponding to the flexible MSM broadband visible light photodetectors numbered 8005, 8010, 8015, and 8020 conductive Ag paste electrodes, respectively. The results indicated the highest sensitivity of the flexible MSM broadband visible light photodetector with 8015 conductive Ag paste electrodes. The photosensitivity of the flexible MSM broadband visible light photodetector was affected by the light transmittance and electrical conductivity of the electrodes. Hence, the highest photosensitivity of the flexible MSM broadband visible light photodetectors indicated better electricity and higher visible light transmittance with the numbered 8015 conductive Ag paste electrode compared to other Ag paste electrodes. Thus, the homemade numbered 8015 conductive Ag paste electrode was suitable for the flexible MSM broadband visible light photodetector. Other related studies for visible light photodetectors are introduced in the following statements: J. B. Yoon et al. fabricated a perfectly aligned CuO nanowire array on a silicon dioxide (SiO_2_) nanograting substrate as a visible light photodetector, and the photosensitivity was 172.21% in the visible light range under 22.5 μW/cm^2^ [45]; T. R. Yew et al. synthesized Co-doped ZnFe_2_O_4_ thin films on a boron-doped silicon substrate as a visible light photodetector, and the photosensitivity was 181 (1.81 × 10^4^%) with 400 °C annealing at a wavelength of 630 nm under 17.9 W/m^2^ [46]. Compared to the reference works, our device reached the same order of visible light photosensitivity under 19.5 W/m^2^. Figure 3c displays the current density of the flexible MSM broadband visible light photodetector with 8015 conductive Ag paste electrodes under different light power densities from 2 W/m^2^ to 20 W/m^2^. The results indicated an increasing photocurrent as the visible light power density increased, indicating increasing photosensitivity. To observe the optical properties of the conductive Ag paste electrodes, a UV/VIS/NIR spectrophotometer was utilized. Figure 4a,b illustrate the reflectance and transmittance of the numbered 8015 conductive Ag paste solution. The results showed that the reflectance of the numbered 8015 conductive Ag paste solution fell between 54.74% and 60.71% in the wavelength range of visible light, from 400 nm to 800 nm, as shown in Figure 4a. Figure 4b shows that the transmittance of the numbered 8015 conductive Ag paste solution varied between 34.83% and 36.98% in the wavelength range of visible light, from 400 nm to 800 nm. The results indicated the semitransparency of the numbered 8015 conductive Ag paste. Hence, the numbered 8015 conductive Ag paste was suitable for the electrodes of flexible MSM broadband visible light photodetectors. The light transmitted through any interface can exhibit the behavior of reflectance, absorption, and transmittance. The reason for the incommensurate transmittance and reflectance curves is due to the absorption.

### 3.3. Flexibility Characteristics of Flexible MSM Broadband Visible Light Photodetectors

**Figure 5:**
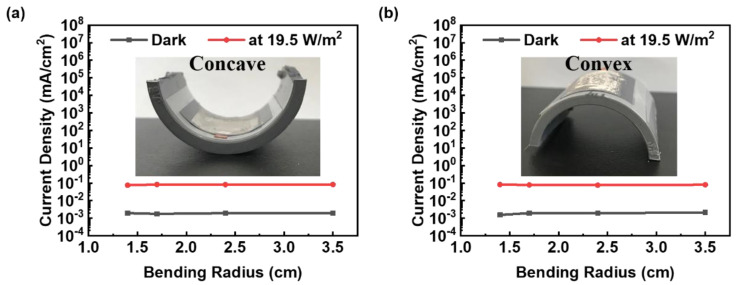
The current density of the flexible MSM broadband visible light photodetector bent with various curvature radii in (**a**) concave and (**b**) convex conditions.

To measure the flexibility of the flexible MSM broadband visible light photodetectors, the photoresponses of bending with different radii were measured. Figure 5a displays the current density of the flexible MSM broadband visible light photodetector bent with various curvature radii in concave conditions. The results indicated that the photocurrent density fell between 7.57 × 10^−2^ mA/cm^2^ and 8.20 × 10^−2^ mA/cm^2^ in concave bending with bending radii of 1.4 cm, 1.7 cm, 2.4 cm, and 3.5 cm. Figure 5b displays the current density of the flexible MSM broadband visible light photodetector bent with various curvature radii under convex conditions. The results indicated that the photocurrent density varied between 7.49 × 10^−2^ mA/cm^2^ and 8.30 × 10^−2^ mA/cm^2^ in convex bending, with bending radii of 1.4 cm, 1.7 cm, 2.4 cm, and 3.5 cm. The photocurrent of the flexible MSM broadband visible light photodetector slightly changed under both concave and convex bending conditions, indicating the stability and durability of our fabricated flexible MSM broadband visible light photodetector devices.

## 4. Conclusions

In summary, we successfully fabricated printable Ag paste electrodes for highly sensitive flexible MSM broadband visible light photodetectors. A flexible Si substrate with a pyramid-like structure was successfully manufactured using a wet etching process. The surficial pyramid-like structure on the flexible Si substrates minimized the reflectance of the incident light, and the average reflectance decreased from 37.06% to 20.76% as a pyramid-like structure was formed. The semitransparent conductive Ag paste electrodes were successfully deposited on flexible Si using a screen-printing process. The reflectance of the homemade numbered 8015 conductive Ag paste solution fell between 54.74% and 60.71%, and the transmittance varied between 34.83% and 36.98% in the wavelength range of visible light, from 400 nm to 800 nm. The highest visible light photosensitivity with the homemade 8015 Ag paste electrode was 1.75 × 10^4^ at 19.5 W/m^2^. The photocurrents of the flexible MSM visible light photodetector were slightly changed under concave and convex conditions, displaying stable and durable bending properties.

## Figures and Tables

**Table 1:** Different numbers correspond to different proportions of homemade silver pastes.

Sample Name	8005	8010	8015	8020
**PU:AgF:H_2_O**	**1.0:4.0:0.5**	**1.0:4.0:1.0**	**1.0:4.0:1.5**	**1.0:4.0:2.0**
**PU:AgF:H_2_O** **(wt %)**	**18:73:9**	**16: 67:16**	**15:62:23**	**14:57:29**

## Data Availability

Not applicable.

## Supplementary Materials

The following supporting information can be downloaded at: https://www.mdpi.com/article/10.3390/nano12142428/s1, Figure S1: Cross-section SEM images of Si substrates during (a) 1 h, (b) 2 h, (c) 3 h, (d) 4 h, (e) 5 h, and (f) 6 h of etching time.
