# Supplementary material for: Screen-Printable Silver Paste Material for Semitransparent and Flexible Metal–Semiconductor–Metal Photodetectors with Liquid-Phase Procedure
Source: Nanomaterials (Basel). 2022 Jul 15;12(14):2428. doi: 10.3390/nano12142428 (PMC9324574; doi:10.3390/nano12142428)
Supplement: Supplementary file 1 [file nanomaterials-12-02428-s001.zip › nanomaterials-1762577-supplementary.pdf]

nanomaterials

Supporting Information

Screen-Printable Silver Paste Material for Semitransparent and Flexible Metal–Semiconductor–Metal Photodetectors with Liquid-Phase Procedure

Shang Yu Tsai [1], Ching-Chang Lin [2], Cheng-Tang Yu [1], Yen-Shuo Chen [1], Wei-Lin Wu [1], Yu-Cheng Chang [3], Chun Chi Chen [4] and Fu-Hsiang Ko [1,*]

[1] Department of Materials Science and Engineering, National Yang Ming Chiao Tung University, 1001 University Road, Hsinchu 30010, Taiwan; ntesst6105@gmail.com (S.Y. T.); tom568899@gmail.com (C.-T.Y.); rubioibur00@gmail.com (Y.-S.C.); lin110489@gmail.com (W.-L.W.)
[2] Research Center for Advanced Science and Technology (RCAST), The University of Tokyo, 4-6-1 Komaba, Meguro-ku, Tokyo 153-8904, Japan; lin@dsc.rcast.u-tokyo.ac.jp
[3] Department of Materials Science and Engineering, Feng Chia University, Taichung 407, Taiwan; yuchchang@fcu.edu.tw
[4] Taiwan Semiconductor Research Institute, 26, Prosperity Road I, Hsinchu Science Park, Hsinchu 300091, Taiwan; chunchi.chen@narlabs.org.tw
* Correspondence: fhko@mail.nctu.edu.tw

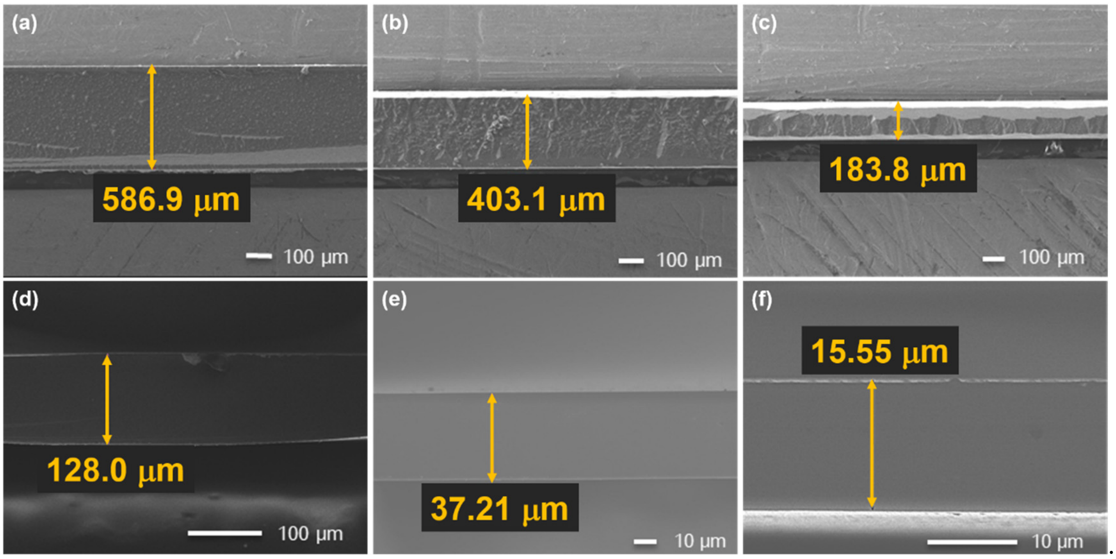

Figure S1. Cross-section SEM images of Si substrates during (**a**) 1 h, (**b**) 2 h, (**c**) 3 h, (**d**) 4 h, (**e**) 5 h, and (**f**) 6 h of etching time.
